# Onset of Regular Smoking Before Age 21 and Subsequent Nicotine Dependence and Cessation Behavior Among US Adult Smokers

**DOI:** 10.5888/pcd17.190176

**Published:** 2020-01-16

**Authors:** Fatma Romeh M. Ali, Israel T. Agaku, Saida R. Sharapova, Elizabeth A. Reimels, David M. Homa

**Affiliations:** 1 Office on Smoking and Health, Centers for Disease Control and Prevention, Atlanta, Georgia; 2 Department of Economics, Faculty of Economics and Political Science, Cairo University, Giza, Egypt

## Abstract

This study assessed the association of regular smoking initiation before age 21 years with nicotine dependence and cessation behaviors among US adult smokers. Data came from the 2014–2015 Tobacco Use Supplement to the Current Population Survey. We found that onset of regular smoking at age 18 to 20 years was associated with higher odds of nicotine dependence and lower odds of attempting and intending to quit. These outcomes were observed with regular smoking initiation at age 18 to 20 as well as before age 18, suggesting that efforts to prevent access to tobacco products before age 21 could reduce nicotine addiction and promote cessation later in life.

**Summary**

**What is already known on this topic?**

Raising the minimum legal age for tobacco sales to 21 years (“T21”) is a promising strategy to reduce tobacco use among youth.

**What is added by this report?**

Adult smokers who started smoking regularly at age 18 to 20 years were more likely to experience high levels of nicotine dependence and less likely to attempt or intend to quit in adulthood compared with those who started at age 21 or older.

**What are the implications for public health practice?**

As part of a comprehensive tobacco control approach, T21 policy could help prevent lifetime addiction to nicotine and promote smoking cessation later in life.

## Objective

Tobacco use is the leading preventable cause of death and disease in the United States (1). Early onset of regular smoking exacerbates risks of smoking-related illnesses and death (2). Raising the minimum legal age for tobacco sales to 21 years (“T21”) is a promising strategy to reduce smoking initiation and thus reduce regular smoking before age 21 (3–5). However, there is limited evidence demonstrating how such policies may reduce nicotine dependence and increase smoking cessation. Previous research examined these outcomes with relation to initiation before age 17 (6,7). This study examined whether delaying regular smoking until after age 21 had additional health benefits beyond those associated with delaying regular smoking until after age 18.

## Methods

Data came from the 2014–2015 Tobacco Use Supplement to the Current Population Survey (TUS-CPS), a nationally representative survey of US adults aged 18 years or older (average response rate, 54.2%; methodology information is available through the US Census Bureau) (8). Analyses were restricted to 25,093 self-reported respondents: 22,070 current smokers who smoked at least 100 cigarettes during their lifetime and 3,023 recent former smokers who quit during the past year, all with information on age at onset of regular smoking and with current age 21 years or older (missing observations, 4.6%).

Age at onset of regular smoking, which was based on responses to the question “How old were you when you first started smoking cigarettes fairly regularly?”, was measured by using 3 indicators: less than 18 years, 18 to 20 years, and 21 years or older.

Nicotine dependence was measured as low, medium, and high by using standardized definitions of the Heaviness of Smoking Index among current daily smokers (composed of time to first cigarette of the day and number of cigarettes smoked per day) (9,10).

Quit attempt was defined as an affirmative response to the question, “During the past 12 months, have you stopped smoking for one day or longer because you were trying to quit smoking?” Recent former smokers also were included to capture all quit attempts. Intention to quit was defined as an affirmative response to the question, “Are you seriously considering quitting smoking within the next 6 months?”

Sociodemographic covariates were sex, current age, race/ethnicity, education, marital status, working status, and annual household income.

Percentages with 95% confidence intervals (CIs) of age at onset of regular smoking and outcomes were reported. Association of age with nicotine dependence was assessed by using ordered logistic regression, whereas associations with quit attempt and intention to quit were assessed by using binary logistic regressions, controlling for sociodemographic covariates and state fixed effects. Analyses were conducted by using Stata version 14 (StataCorp LLC) and weighted to yield nationally representative estimates.

## Results

### Prevalence of age at onset of regular smoking and outcomes

Prevalence of onset of regular smoking before age 18, at age 18 to 20, and at age 21 or older were 50.1%, 33.1%, and 16.8%, respectively (Table 1). Demographic characteristics varied significantly (*P* < .05) across the 3 groups, with higher proportions of men and non-Hispanic whites among adults who started regular smoking before age 18 or at age 18 to 20 compared with those who started at age 21 or older. The proportion of those with a college degree or higher was lower among adult smokers who started regular smoking before age 18 compared with the other age groups.

**Table 1:** Characteristics of Adult Smokers by Age at Onset of Regular Smoking^a^, United States, 2014–2015^b^

Characteristics	% Onset of Regular Smoking at Age <18 (95% CI)	% Onset of Regular Smoking at Age 18–20 (95% CI)	% Onset of Regular Smoking at Age ≥21 (95% CI)
**Overall**	50.1 (49.6–50.6)	33.1 (32.6–33.6)	16.8 (16.4–17.1)
**Sex**			
Female	43.1 (42.4–43.8)	44.9 (44.0–45.8)	50.1 (48.9–51.3)
Male	56.9 (56.2–57.6)	55.1 (54.2–56.0)	49.9 (48.7–51.1)
**Current age, y**			
21–24	5.6 (5.2–6.0)	6.7 (6.1–7.3)	1.8 (1.4–2.3)
25–44	31.6 (30.9–32.3)	31.3 (30.4–32.1)	27.5 (26.4–28.6)
45–64	40.6 (39.9–41.3)	36.2 (35.4–37.1)	42.3 (41.1–43.5)
≥65	22.2 (21.6–22.7)	25.8 (25.0–26.5)	28.4 (27.3–29.4)
**Race/ethnicity**			
White, non-Hispanic	78.5 (77.8–79.1)	76.7 (75.9–77.5)	67.7 (66.5–68.9)
Black, non-Hispanic	8.0 (7.6–8.4)	8.5 (8.0–9.0)	14.0 (13.1–14.8)
Other, non-Hispanic	4.4 (4.1–4.8)	6.0 (5.5–6.5)	7.5 (6.7–8.2)
Hispanic	9.1 (8.7–9.6)	8.8 (8.2–9.4)	10.9 (10.1–11.8)
**Education**			
Less than high school graduate	15.8 (15.3–16.3)	8.3 (7.8–8.8)	10.3 (9.6–11.1)
High school graduate	36.1 (35.4–36.8)	30.3 (29.5–31.1)	29.5 (28.4–30.6)
Some college	31.1 (30.5–31.8)	33.2 (32.4–34.0)	32.9 (31.8–34.0)
College graduate or higher	17.0 (16.4–17.5)	28.2 (27.4–28.9)	27.3 (26.2–28.4)
**Marital status**			
Unmarried	51.3 (50.6–52.0)	48.7 (47.8–49.6)	54.6 (53.4–55.8)
Married	48.7 (48.0–49.4)	51.3 (50.4–52.2)	45.4 (44.2–46.6)
**Working status**			
Nonworking	45.7 (45.0–46.4)	41.7 (40.8–42.6)	45.7 (44.5–46.9)
Working	54.3 (53.6–55.0)	58.3 (57.4–59.2)	54.3 (53.1–55.5)
**Annual household income, $**			
<25,000	30.3 (29.6–31.0)	23.2 (22.4–23.9)	28.9 (27.8–30.0)
25,000–49,999	28.2 (27.6–28.8)	26.9 (26.1–27.7)	27.6 (26.5–28.7)
50,000–74,999	17.6 (17.1–18.1)	19.6 (18.9–20.3)	17.5 (16.6–18.4)
≥75,000	23.9 (23.3–24.5)	30.3 (29.5–31.1)	26.1 (25.0–27.1)

Abbreviation: CI, confidence interval.

a This was defined based on respondents’ answer to the survey question “How old were you when you first started smoking cigarettes fairly regularly?”, categorized as <18 years, 18–20 years, and ≥21 years.

b Significance between subgroups was assessed by using χ^2^ test. Significant differences (*P* < .05) were observed among all subgroups listed in the tables.

Overall, 49.5% made at least 1 quit attempt in the previous 12 months. Among current smokers, 46.2% were considering quitting cigarette smoking seriously within the next 6 months. Among current daily smokers, prevalence of low, medium, and high nicotine dependence was 30.2%, 64.6%, and 5.2%, respectively.

### Association between age at onset of regular smoking and outcomes

The adjusted odds of high nicotine dependence (vs combined medium and low nicotine dependence) were greater among those who started smoking regularly under age 18 than those who started at age 21 or older (adjusted odds ratio [AOR], 2.15; 95% CI, 1.92–2.39) (Table 2). Onset of regular smoking at age 18 to 20 also was associated with higher nicotine dependence in adulthood than at age 21 or older (AOR, 1.25; 95% CI, 1.11–1.41).

**Table 2:** Adjusted Odds Ratios of Nicotine Dependence and Cigarette Smoking Cessation Among US Adult Smokers, 2014–2015^a^

Characteristics	AOR of High Nicotine Dependence^b^ (95% CI)	AOR of Quit Attempt^c^ (95% CI)	AOR of Intention to Quit^d^ (95% CI)
**Age at onset of regular smoking, y**			
≥21	1 [Reference]	1 [Reference]	1 [Reference]
18–20	1.25 (1.11–1.41)	0.83 (0.75–0.90)	0.73 (0.66–0.81)
<18	2.15 (1.92–2.39)	0.75 (0.69–0.81)	0.66 (0.60–0.72)
**Sex**			
Female	1 [Reference]	1 [Reference]	1 [Reference]
Male	1.47 (1.36–1.60)	0.89 (0.84–0.95)	0.88 (0.82–0.94)
**Current age, y**			
21–24	1 [Reference]	1 [Reference]	1 [Reference]
25–44	1.75 (1.47–2.10)	0.70 (0.61–0.81)	1.06 (0.91–1.24)
45–64	2.56 (2.14–3.07)	0.51 (0.44–0.59)	0.97 (0.83–1.13)
≥65	2.14 (1.74–2.64)	0.45 (0.38–0.53)	0.68 (0.57–0.82)
**Race/ethnicity**			
White, non-Hispanic	1 [Reference]	1 [Reference]	1 [Reference]
Black, non-Hispanic	0.47 (0.41–0.54)	1.17 (1.05–1.30)	1.29 (1.15–1.44)
Other, non-Hispanic	0.65 (0.54–0.79)	1.07 (0.92–1.24)	0.91 (0.78–1.07)
Hispanic	0.29 (0.24–0.34)	1.17 (1.04–1.33)	1.11 (0.97–1.27)
**Education**			
Less than high school graduate	1 [Reference]	1 [Reference]	1 [Reference]
High school graduate	0.86 (0.76–0.97)	1.07 (0.97–1.17)	1.02 (0.92–1.13)
Some college	0.75 (0.66–0.85)	1.33 (1.20–1.46)	1.23 (1.10–1.36)
College graduate or higher	0.56 (0.47–0.66)	1.38 (1.22–1.56)	1.25 (1.10–1.43)
**Marital status**			
Unmarried	1 [Reference]	1 [Reference]	1 [Reference]
Married	0.90 (0.83–0.98)	1.11 (1.04–1.19)	1.06 (0.99–1.14)
**Working status**			
Nonworking	1 [Reference]	1 [Reference]	1 [Reference]
Working	0.84 (0.77–0.92)	0.90 (0.84–0.97)	0.95 (0.88–1.02)
**Annual household income, $**			
<25,000	1 [Reference]	1 [Reference]	1 [Reference]
25,000–49,999	0.87 (0.79–0.97)	0.99 (0.91–1.07)	0.93 (0.85–1.01)
50,000–74,999	0.84 (0.74–0.95)	0.99 (0.90–1.09)	0.88 (0.79–0.97)
≥75,000	0.79 (0.69–0.90)	1.05 (0.95–1.17)	1.08 (0.97–1.21)
**State fixed effects**	Yes	Yes	Yes
**Sample size**	16,865	24,648	21,504

Abbreviations: AOR, adjusted odds ratio; CI, confidence interval.

a Significance was set at *P* < .05, Wald test.

b Nicotine dependence was defined for current daily smokers based on the Heaviness of Smoking Index (HSI; low, moderate, and high) (9). The HSI index was calculated from the number of cigarettes smoked per day and time to the first cigarette of the day. The model was estimated by using an ordered logistic regression model adjusted for all covariates listed in the table.

c This includes both current smokers who were asked “During the past 12 months, have you stopped smoking for one day or longer because you were trying to quit smoking?” and recent former smokers who quit cigarette smoking within the previous 12 months. The model was estimated by using a binary logistic regression model adjusted for all covariates listed in the table.

d This includes current smokers who were asked “Are you seriously considering quitting smoking within the next 6 months?” The model was estimated by using a binary logistic regression model adjusted for all covariates listed in the table.

Those who started smoking regularly before age 18 had smaller adjusted odds of quit attempt (AOR, 0.75; 95% CI, 0.69–0.81) and intention to quit (AOR, 0.66; 95% CI, 0.60–0.72) than those who started smoking at age 21 or older. Starting regular smoking at age 18 to 20 also was associated with smaller odds of quit attempt (AOR, 0.83; 95% CI, 0.75–0.90) and intention to quit (AOR, 0.73; 95% CI, 0.66–0.81) than starting at age 21 or older.

## Discussion

The findings of this study suggest that starting smoking regularly at age 18 to 20 was associated with higher odds of nicotine dependence and lower odds of smoking cessation than starting at age 21 or older. These negative health consequences were observed with regular smoking initiation at age 18 to 20 as well as before age 18. Therefore, efforts to prevent regular smoking initiation before age 21 could help prevent lifetime addiction to nicotine and promote smoking cessation later in life.

T21 policies aim to prevent youth access to tobacco products before age 21, reducing the likelihood of regular smoking initiation before this age. Although national empirical evidence about the impact of T21 is limited, evidence from local jurisdictions show that T21 is an effective strategy to limit the ability of youths to purchase tobacco products and to reduce the youth smoking rate (4,5). Additionally, T21 is supported by most US adults, including 7 in 10 smokers (11).

This study has some limitations. First, all data were self-reported and may be subject to recall error. Second, the exact age when an individual became a regular smoker might not be precisely determined, as progression from experimental to regular smoking might have transitioned between various patterns of experimentation, quitting, relapses, and regular smoking. Third, age at which a respondent first smoked a cigarette is unknown and thus implications of this study for T21 policies should be interpreted with caution.

As the tobacco product landscape continues to evolve and new and emerging products enter the market, it is critical that T21 policies keep pace by including the diversity of tobacco products being sold in the United States, including e-cigarettes. It will also be important to continue to monitor future enactment of T21 policies at the state and local levels and track the impact on youth smoking initiation and adult smoking prevalence over time.
